# Comparative transcriptome analysis of eggplant (*Solanum melongena* L.) and turkey berry (*Solanum torvum* Sw.): phylogenomics and disease resistance analysis

**DOI:** 10.1186/1471-2164-15-412

**Published:** 2014-05-31

**Authors:** Xu Yang, Yu-Fu Cheng, Cao Deng, Yan Ma, Zhi-Wen Wang, Xue-Hao Chen, Lin-Bao Xue

**Affiliations:** College of Horticulture and Plant Protection of Yangzhou University, Yangzhou, 225009 China; PubBio-Tech Services Corporation, Wuhan, 430070 China

**Keywords:** *Solanum torvum* Sw., *Solanum melongena* L., Comparative transcriptomics, Evolution, Plant resistance genes

## Abstract

**Background:**

Eggplant (*Solanum melongena* L*.*) and turkey berry (*S. torvum* Sw.), a wild ally of eggplant with promising multi-disease resistance traits, are of great economic, medicinal and genetic importance, but genomic resources for these species are lacking. In the present study, we sequenced the transcriptomes of eggplant and turkey berry to accelerate research on these two non-model species.

**Results:**

We built comprehensive, high-quality *de novo* transcriptome assemblies of the two *Leptostemonum* clade *Solanum* species from short-read RNA-Sequencing data. We obtained 34,174 unigenes for eggplant and 38,185 unigenes for turkey berry. Functional annotations based on sequence similarity to known plant datasets revealed a distribution of functional categories for both species very similar to that of tomato. Comparison of eggplant, turkey berry and another 11 plant proteomes resulted in 276 high-confidence single-copy orthologous groups, reasonable phylogenetic tree inferences and reliable divergence time estimations. From these data, it appears that eggplant and its wild *Leptostemonum* clade relative turkey berry split from each other in the late Miocene, ~6.66 million years ago, and that *Leptostemonum* split from the *Potatoe* clade in the middle Miocene, ~15.75 million years ago. Furthermore, 621 and 815 plant resistance genes were identified in eggplant and turkey berry respectively, indicating the variation of disease resistance genes between them.

**Conclusions:**

This study provides a comprehensive transcriptome resource for two *Leptostemonum* clade *Solanum* species and insight into their evolutionary history and biological characteristics. These resources establish a foundation for further investigations of eggplant biology and for agricultural improvement of this important vegetable. More generally, we show that RNA-Seq is a fast, reliable and cost-effective method for assessing genome evolution in non-model species.

**Electronic supplementary material:**

The online version of this article (doi: 10.1186/1471-2164-15-412) contains supplementary material, which is available to authorized users.

## Background

Eggplant (*Solanum melongena* L.) is the third most agriculturally important crop from the genus *Solanum* after potato (*S. tuberosum*) [1] and tomato (*S. lycopersicum*) [2]. This large and diverse genus of flowering plants comprises >1400 species having a wide range of genetic and phenotypic variation [3]. In 2011, 46.8 million tons of eggplant was produced in the top four producing countries, namely China (27.7 million tons), India (11.8 million tons), Egypt (1.1 million tons) and Turkey (8.2 million tons), according to the Food and Agriculture Organization of the United Nations (http://faostat.fao.org). There are three closely related cultivated species of eggplant, all of Old World origin: *S. aethiopicum* L. (scarlet eggplant), *S. macrocarpon* L. (gboma eggplant) and *S. melongena* L. (brinjal or aubergine eggplant) [4]. The brinjal or aubergine eggplant, hereafter referred to as eggplant, is cultivated worldwide and is an autogamous diploid with 12 chromosomes (2n = 2x = 24) [5]. Eggplant is susceptible to many bacterial and fungal pathogens and insects, such as the *Verticillium dahlia* fungus and nematodes [6], which cause significant yield losses. As such, improving resistance to biotic and abiotic stresses is one of the main objectives of eggplant breeding programs.

*Solanum torvum* Sw., commonly known as turkey berry, is a wild relative of eggplant and is found in tropical Africa, Asia and South America. Turkey berry is widely consumed and is an important folk medicinal plant in tropical and subtropical countries [7]. More importantly, turkey berry is resistant to root-knot nematodes and the most serious soil-borne diseases, such as those caused by *Ralstonia solanacearum*, *V. dahlia* Klebahn and *Fusarium oxysporum* f. sp. Melongenae [8], providing promising genetic resources for improvement of eggplant. Traditional grafting techniques are now used worldwide in eggplant cultivation, in which eggplant tissues are grafted onto disease-resistant rootstock of turkey berry [8–10]. Also, attempts have been made to introduce turkey berry resistance into eggplant through conventional breeding and biotechnological techniques, however, progress is limited. Owing to sexual incompatibilities, however, attempts at crossing eggplant with turkey berry have had limited success [11], and sterile hybrids were obtained, with difficulty, only when eggplant was used as the female parent [12]. Other biotechnological techniques, such as embryo rescue, somatic hybridization and Agrobacterium-mediated transformation, have been difficult to apply to eggplant [12, 13] because of the limited genetic information available for this species.

*Solanum* crops that belong to the *Potatoe* clade, which includes potato and tomato, have been targets for comprehensive genomic studies [1, 2]. However, genomic resources are lacking for the *Leptostemonum* clade (the “spiny solanums”), which comprises almost one-third of the genus distributed worldwide [14] and includes eggplant and turkey berry. For eggplant, 98,861 nucleotide sequences have been deposited in the National Center for Biotechnology Information (NCBI) GenBank database (as of December 18, 2013), and the vast majority of them (98,086) were provided recently by a comparative analysis of ESTs [15]. In that analysis, however, only 16,245 unigenes were constructed, which is approximately half the number of genes identified in the closely related potato (39,031) [1] and tomato (34,727) [2], implying that these unigenes represent only a limited portion of the whole eggplant transcriptome. In addition, large numbers of short-read sequences have been generated from turkey berry in attempts to identify single nucleotide polymorphisms and simple sequence repeats using restriction site–associated DNA tag sequencing strategies; however, this approach provides only limited information on full-length genes, and such information is vital for identifying trait-related genes and for quantitative gene expression analysis. Recent studies reported 6,296 unigenes from *S. torvum* cultivar Torubamubiga [8] and 36,797 unigenes from *S. torvum* Sw. accession TG1 transcriptome assemblies [16]. In the latter study, however, sequencing was confined to the 3′ end of the transcripts, resulting in fragmentary assembled transcripts as revealed by an N50 value (the 50% of the entire assembly is contained in sequences equal to or larger than this value) of only 514 bp and an N10 value of only 715 bp. Therefore, there is an urgent need to obtain more high-quality genomic information about eggplant and turkey berry, and a promising technology to accomplish this is RNA sequencing (RNA-Seq).

High-quality transcriptome data would not only facilitate genetic and molecular breeding approaches in eggplant and allow genomic resource mining in turkey berry but also be valuable for comparative biology studies, such as phylogenomics. For example, RNA-Seq data have been used to explore the evolution of paleopolyploidy in plants [17, 18] and to reconstruct deep phylogenies in flowering plants of the grape family (Vitaceae) [19]. These studies suggest that transcriptome data can be very useful and practical in the reconstruction of phylogenies in flowering plants.

The specific goals of this study were to (1) generate high-quality transcripts and unigenes of eggplant and turkey berry using RNA-Seq, which will provide reference transcriptomes for further analysis, such as trait-related gene mining and quantitative expression analysis; (2) produce a dated phylogeny of the *Potatoe* and *Leptostemonum* clades and of the *Leptostemonum*-nested eggplant (Old Word clade) and turkey berry (Torva clade), which will deepen our understanding of phylogenetic relationships and ultimately assist crop improvement; and (3) identify and compare disease resistance genes in eggplant and turkey berry to take a first glance at the variation of resistance genes among them using RNA-Seq data.

## Results and discussion

### *De novo* transcriptome assembly and annotation captures high-quality transcripts and unigenes

**Figure 1:**
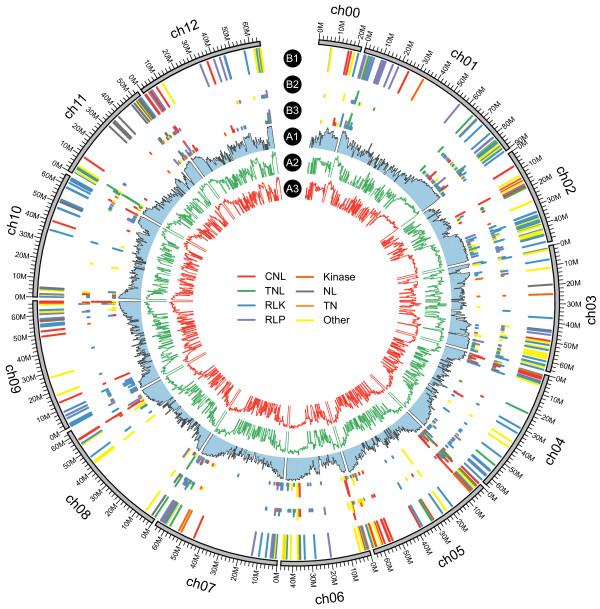
**Distributions of genomic elements of tomato, eggplant and turkey berry on tomato genome. A1**, The log2-transformed tomato gene density (blue histogram ring) along the tomato chromosomes (ch, outer circle). Gene density represented as number of genes per 500 kb (non-overlapping, window size = 500 kb), and the log2-transformed gene density ranged from 0.00 to 6.50. **A2** and **A3**, The log10-transformed average depth of RNA-Seq reads from eggplant (**A2**, green histogram ring) and turkey berry (**A3**, red histogram ring). We used the 500kp non-overlapping sliding windows to calculated the average depth, and the log10-transformed average depth ranged from 1.50 to 6.50. **B1**, Tomato resistance genes. Colors correspond to the gene product types indicated in the center of the diagram. **B2** and **B3**, resistance genes of eggplant (B2 ring) and turkey berry (B3 ring). The square root of the number of resistance genes per tomato homolog (BLASTX hits) ranged from 1.00 to 3.00 (for illustration purposes, the minimum was set at 0.80).

To maximize the range of transcript diversity and completeness, mixed RNA samples from three tissues of each plant were prepared for Illumina sequencing. We obtained 2.24 Gb and 3.94 Gb of sequence from eggplant and turkey berry respectively (Table 1), and the raw paired-end data were deposited in the NCBI Sequence Read Archive. The cleaned reads were aligned to the genomes of the closely related *Solanum* species tomato and potato to assess sequencing completeness. As shown in Figure 1 (rings A1–A3), the depth distribution of eggplant and turkey berry fit well to the tomato gene distribution. Similarly, the eggplant and turkey berry reads fit well with the potato gene distribution (Additional file 1: Figure S1, rings A1–A3). These results indicate that the sequencing reads obtained from eggplant and turkey berry covered the majority of genes in these species.

**Table 1:** **Summary of the eggplant and turkey berry transcriptome assemblies**

		Turkey berry	Eggplant
Total raw reads		27,387,245 × 2	15,576,018 × 2
Read length		72 + 72	72 + 72
Total raw reads data size (bp)		3,943,763,280	2,242,946,592
GC (%)		44.36	44.48
Contigs	number	953,817	388,048
	total length	94,028,534	54,207,749
	N50	80	275
	max length	10,665	12,935
Transcripts	number	53,596	44,672
	total length	49,514,233	40,664,371
	N50	1,481	1,445
	max length	10,684	12,935
Unigenes	number	38,185	34,174
	total length	30,868,727	27,771,410
	N50	1,349	1,326
	max length	10,684	12,935

**Figure 2:**
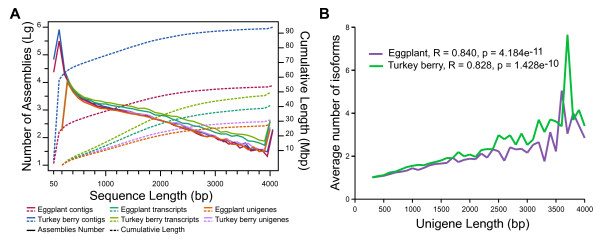
**Length distribution of contigs, transcripts and unigenes of eggplant and turkey berry. A**, Distribution of assemblies (contigs, transcripts and unigenes). The left y-axis and solid lines are the distributions of number (log10-transformed) of assemblies in each 100-bp bin, while the right y-axis and dashed lines are the cumulative curves for each assembly. **B**, Distributions of average numbers of isoforms in each bin (100 bp). Pearson's correlation coefficient tests were carried out using the *cor.test* function in R version 3.0.1.

Clean reads from the two *Solanum* species were then separately assembled into contigs and clustered into transcripts using the *de novo* transcriptome assembler Trinity, which can efficiently reconstruct full-length transcripts across a broad range of expression levels and sequencing depths [20]. The clustering step substantially improved the assembly quality, as indicated by elevated N50 values and decreased total length, by eliminating redundant contigs (Table 1 and Figure 2A). Similar transcripts in the same cluster are thought to be isoforms (splice variants) at the gene locus [20]. To further eliminate redundant transcripts and to obtain the primary representative of each gene locus, only the longest transcript in each cluster was regarded as the final assembled unigene. This process identified 34,174 unigenes for eggplant and 38,185 unigenes for turkey berry (Table 1), which included 9,743 (28.51%) and 10,762 (28.18%) unigenes longer than 1 kb respectively. We observed a decrease in N50 values of unigenes compared with transcripts, suggesting that longer genes may tend to generate more isoforms. This hypothesis was confirmed by plotting unigene length against the average number of isoforms in each bin and performing a Pearson's correlation coefficient test (Figure 2B), which showed a significant positive correlation for both eggplant and turkey berry.

To evaluate the completeness of our assemblies, the transcripts and unigenes were aligned with the tomato and potato sequences to obtain the corresponding reference genes, and then the unigene and transcript distributions were plotted against the tomato and potato reference genomes. The unigene and transcript distribution patterns were similar to the gene distribution patterns of both the tomato (Additional file 1: Figure S2) and potato (Additional file 1: Figure S3), indicating the completeness of the unigene assemblies.

Our assemblies were of substantially higher quality than those generated in previous studies [15, 16]. In a comparative analysis of eggplant ESTs [15], only 16,245 unigenes were constructed, which is less than half of our 34,174 unigenes and of the genes identified in the closely related potato (39,031) [1] and tomato (34,727) [2]. Global transcriptome profiling aimed at gaining insight into the mechanisms underpinning turkey berry resistance against *Meloidogyne incognita*[16] produced 36,797 unigenes from *S. torvum* Sw. accession TG1. Although this number is comparable to our results, to improve coverage and conserve specificity, sequencing in that study was confined to the 3′ end of the transcripts, resulting in a fragmented assembly, as indicated by low N50 (514 bp) and N10 (715 bp) values. Without introduced bias, our N50 value was 1,349 bp, which is similar to the N50 of the non-redundant coding sequences (CDS) from tomato (1,467 bp) and potato (1,257 bp). Taken together, these results suggest that the quality and completeness of our sequencing and assembly were high enough for annotation and further analyses.

Annotation provides important information on gene function and structure. We were able to annotate 81.98% (28,016) of the eggplant unigenes and 78.16% (29,845) of the turkey berry unigenes with a threshold of 1e^–5^ by performing a BLASTX search against diverse protein databases. When we extracted and aligned the putative CDSs, 86.96% (29,717) of eggplant unigenes and 84.03% (32,086) of turkey berry were annotated (Table 2). These results further confirmed the high quality of the *de novo* assembly.

**Table 2:** **Annotation results of the eggplant and turkey berry unigenes**

		Turkey berry		Eggplant	
		Number	Percentage	Number	Percentage
Functional annotations	Total	29,845	78.16%	28,016	81.98%
	NR	29,072	76.13%	27,393	80.16%
	*Solanum*	29,571	77.44%	27,846	81.48%
	SwissProt	17,269	45.22%	16,021	46.88%
	KEGG	14,666	38.41%	13,754	40.25%
	COG	9,089	23.80%	8,419	24.64%
	GO	17,890	46.85%	16,982	49.69%
CDS annotations	Total	32,086	84.03%	29,717	86.96%
	Homolog	27,849	72.93%	26,251	76.82%
	ESTScan	406	1.06%	278	0.81%
	HMM	3,831	10.03%	3,188	9.33%

CDS: coding sequence, NR: NCBI non-redundant protein database, *Solanum*: potato (PGSC DM 3.4) and tomato (ITAG2.3) genomes, KEGG: Kyoto Encyclopedia of Genes and Genomes, COG: NCBI clusters of orthologous groups database, GO: gene ontology determined by BLAST2GO, Homolog: CDS annotated with homologous approach, ESTScan: CDS annotated by ESTScan software, HMM: CDS modeled by fifth-order HMM (hidden Markov Model).

**Figure 3:**
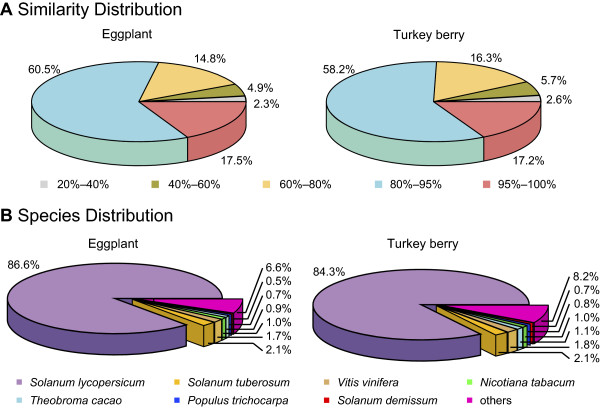
**Similarity and species distribution of the top hits in the NCBI NR database.**

In a BLASTX homolog search against the NCBI non-redundant (NR) protein database, 27,393 eggplant unigenes and 29,072 turkey berry unigenes had matches (Table 2), 78.0% and 75.4% respectively, of which showed >80% identity (Figure 3A), indicating the high accuracy of the assembly. For both species, the top hit species was tomato, followed by potato and then grape (*Vitis vinifera*) (Figure 3B). Interestingly, only 2.1% of the top hits were assigned to potato*,* which is much less than the 86.6% of eggplant and 84.3% of turkey berry hits that were assigned to tomato. A similar result was observed in an EST-based comparative analysis of eggplant [15], suggesting that these two species are more closely related to tomato than potato.

### Comparative analysis of gene sets between plants

**Figure 4:**
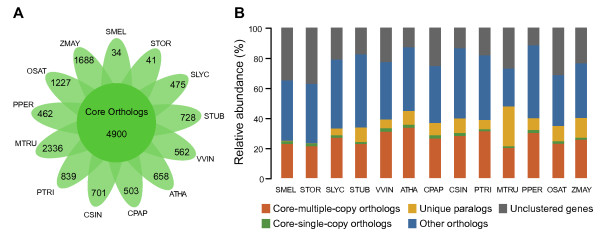
**Orthologous group analysis of 13 species. A**, Flower plot showing the numbers of orthologous groups in which only specific species are present (petals) and the number of core orthologous groups in which all species are present (center). **B**, Spinogram depicting the composition of different categories of orthologous groups. SMEL, *S. melongena* L.; STOR, *S. torvum* Sw.; SLYC, *S. lycopersicum*; STUB, *S. tuberosum*; ATHA, *A. thaliana*; CPAP, *C papaya*; VVIN, *V. vinifera*; PTRI, *P. trichocarpa*; PPER, *P. persica*; CSIN, *C. sinensis*; MTRU, *M. truncatula*; ZMAY, *Z. mays*; OSAT, *O. sativa* japonica.

A total of 427,731 proteins from eggplant (29,717), turkey berry (32,086) and 11 other plant species, including tomato, potato, *Arabidopsis thaliana*, *Carica papaya*, *V. vinifera*, *Prunus persica*, *Citrus sinensis*, *Medicago truncatula*, *Zea mays* and *Oryza sativa* japonica, were binned into 36,627 orthologous groups (gene families) using OrthoMCL v2.0.9 [21] following self-self-comparison with the BLASTP program. The average number of genes in each gene family (Table 3), the number of unique gene families (Figure 4A), and number of genes in the unique gene families (Figure 4B) of eggplant and turkey berry were less than those of tomato, potato and other plants. This suggests that either eggplant and turkey berry have distinct gene family features or that our gene sets are incomplete. Although our RNA libraries were derived from mixed tissue samples, it is likely that not all genes in the genome are represented in our transcriptomes.

**Table 3:** **Summary of orthologous groups between 13 species**

Species	Number of genes	Unclustered	Genes in families	Number of families	Average genes per family
*S. melongena* L.	29,717	10,407	19,310	15,421	1.252
*S. torvum* Sw.	32,086	11,989	20,097	16,069	1.251
*S. lycopersicum*	33,585	7,135	26,450	16,870	1.568
*S. tuberosum*	38,492	6,791	31,701	16,586	1.911
*V. vinifera*	25,329	5,784	19,545	13,080	1.494
*A. thaliana*	26,637	3,479	23,158	12,944	1.789
*C. papaya*	25,599	6,552	19,047	13,398	1.422
*C. sinensis*	28,767	3,950	24,817	14,171	1.751
*M. truncatula*	43,683	11,858	31,825	12,741	2.498
*P. persica*	27,792	3,232	24,560	14,152	1.735
*P. trichocarpa*	40,984	7,533	33,451	14,912	2.243
*O. sativa* japonica	35,402	11,163	24,239	15,392	1.575
*Z. mays*	39,658	9,412	30,246	15,821	1.912

Nevertheless, 4,900 orthologous groups were shared by all 13 species (Figure 4A), which is comparable to previous studies. Wang *et al.*[22] found 9,525 shared core orthologous groups between *Gossypium raimondii*, *Theobroma cacao*, *A. thaliana* and *Z. mays*, D’Hont *et al.*[23] found 7,674 shared gene families between *Musa acuminata*, *Phoenix dactylifera*, *A. thaliana*, *O. sativa*, *Sorghum bicolor* and *Brachypodium distachyon*, and Peng *et al*. [24] found 9,451 shared gene families among five grass genomes. The numbers of orthologous groups that we observed were smaller, but the groups included more species, which may indicate that our analysis was more stringent and therefore may represent only highly conserved orthologous groups among dicotyledonous and monocotyledonous plants. Among the 4,900 core orthologous groups, 559 contained only one ortholog in each species (single copy, Figure 4B). These groups were suitable for inferring phylogenetic relationships and for estimating divergence time.

### Inferring phylogenetic relationships

To maximize the information content of our sequences and minimize the impact of missing data, the 559 single-copy orthologous groups were further filtered with stricter constraints on length (minimum 200 amino acids) and sequence alignment (maximum missing data 50% in the CDS alignments), and the resultant 276 groups were used for phylogenetic tree reconstruction.

**Figure 5:**
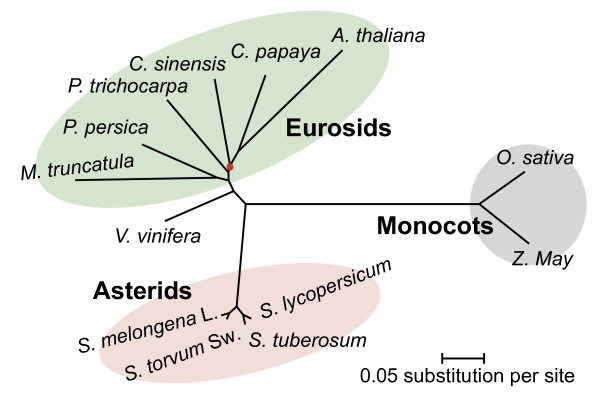
**Maximum likelihood unrooted tree based on the second-codon positions of 276 single-copy genes.** All of the nodes have 100% bootstrap support values except the node marked with the red dot, which has a bootstrap value of 88%.

The CDS alignments from the 276 refined single-copy orthologous groups were first concatenated to form one supergene for each species, each of which was then subjected to phylogenetic analyses with the maximum likelihood method in PhyML3.1 [25]. Unexpectedly, the phylogenies obtained (Additional file 1: Figure S4A) were incongruent with the well-recognized Angiosperm Phylogeny Group III (APG III) system [26]. Notably, the branch lengths (indicating substitutions per site) varied considerably in our tree, indicating relatively variable evolution rates among species. Quite different substitution rates are commonly observed for the three positions within codons, with the third position being especially variable as a result of the degeneracy of the genetic code. Third-position substitutions are likely to be saturated and may accumulate mutational bias, which may influence the accuracy of phylogeny estimations [27]. Therefore, the CDS alignments of each of the 276 gene families were separated into three datasets corresponding to each of the three codon positions in the CDS, and another three supergenes were assembled and used to estimate phylogeny. As predicted, the three maximum likelihood trees were identical (Figure 5 and Additional file 1: Figure S4B–D) and placed the monocot, Asterids, grape and Eurosids clades in accordance with the APG III system. Notably, all the clades leading to Asterid species had 100% bootstrap support values, even in the uncorrected tree (Additional file 1: Figure S4), implying that the RNA-Seq assemblies may not be responsible for the incongruence of phylogenies that we observed when using full-length CDS sequences and also providing robust support for the known relationships in Asterid species. As shown in Figure 5, eggplant was most closely related to its *Leptostemonum* clade relative turkey berry, and further separated from the members of the *Potatoe* clade, tomato and potato [14, 15].

### Estimation of divergence time

**Figure 6:**
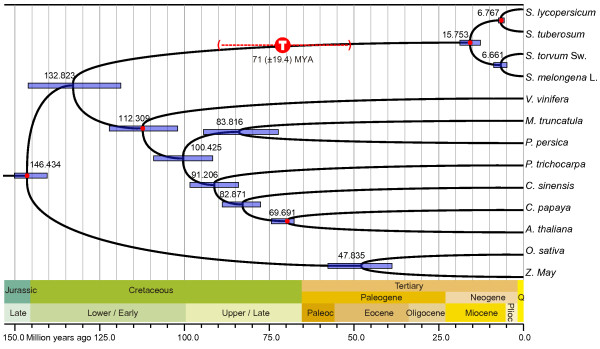
**Estimation of divergence time using the three codon position–specific datasets.** The purple bars at the nodes indicate 95% posterior probability intervals. The geological time scale is in millions of years. The red dots correspond to the calibration time points listed in the Materials and Methods. Confirmed whole-genome triplication shared by *Solanum* and estimated at 71 (±19.4) MYA [2] is shown with annotated circles (‘T’), with dashed line indicating confidence interval. Paleoc, Paleocene; Plioc, Pliocene; Q, Quaternary.

The three codon position–based supergene sets from the 276 single-copy orthologous groups were used for combination analysis of multi-partitions in the MCMCTree program (PAML4.7 package) [28]. The same substitution model was used, but different parameters were assigned and estimated for each set. Moreover, because of the variable evolution rate among species we observed, the clock model with independent rates among lineages specified by a log-normal probability distribution was adopted [29]. To check the robustness of results, we ran the MCMCTree analysis twice and obtained similar results, and a chronogram (Figure 6) was produced using FigTree v1.4.0 (http://tree.bio.ed.ac.uk/) from the first run. Another dataset containing only the first two supergene sets (after removing the fast-evolving third position) was subjected to MCMCTree analysis, and a similar chronogram was obtained (Additional file 1: Figure S5).

All of the geological times estimated for nodes leading to non-Asterid species were well matched to data deposited in TimeTree [30], a public knowledge-base of divergence times among organisms, demonstrating the high reliability of this molecular clock dating strategy. As shown in Figure 6, the divergence between eggplant and turkey berry appears to have occurred ~6.66 (4.9–8.8) million years ago (Mya), during the late Miocene. The *Leptostemonum* and *Potatoe* clades shared a common ancestor during the middle Miocene and appear to have diverged ~15.75 (12.7–18.8) Mya, which is in agreement with the 11.60–16.00 Mya estimated by Wang *et al.*[31]. A whole-genome triplication in tomato [2] and potato [1] has been estimated at 71 (±19.4) Mya on the basis of synonymous substitutions of paralogous genes, which is much earlier than the splitting of *Leptostemonum* and *Potatoe* clades. This timeline implies, therefore, that both eggplant and turkey berry underwent genome triplication, but this remains to be verified by complete genome sequences.

### Disease resistance genes

A fundamental strategy for controlling diseases in agriculturally important plants is the isolation of resistance genes from their less susceptible relatives to be used in conventional breeding, genetic engineering and biotechnological approaches [12, 13]. Because of limited genetic resources for eggplant and turkey berry, however, only one resistance gene, a *Ve-*like gene (*StVe*), has been identified in these species, to our knowledge [32]. Moreover, a large number of plant resistance genes have been identified and deposited in the Plant Resistance Genes database (PRGdb, http://prgdb.crg.eu/wiki/Main_Page) [33]. Of these entries, 112 were manually curated to confirm that they were described in the literature to confer resistance to pathogens, and they are grouped into seven distinct classes based on the presence of specific domains or partial domains [34, 35]: N-terminal coiled coil–nucleotide-binding site–leucine-rich repeat (CNL), Toll interleukin1 receptor–nucleotide-binding site–leucine-rich repeat (TNL), receptor-like kinase (RLK), receptor-like protein (RLP), three truncated classes (Kinase, NL and TN) and ‘Other’ which has no typical resistance related domains. Of the 112 entries, 36 (32.14%) are from Solanaceae, 37 (33.04%) are from Poaceae, 25 (22.32%) are from Brassicaceae, and only 14 (12.50%) are from other families. The high percentage of closely related sequences (from Solanaceae) and outgroup sequences (from monocot, Poaceae) made it possible to identify and classify both recently arisen and ancient orthologous resistance genes through homology-based approaches.

Amino acid sequences for the 112 reference resistance genes were downloaded from the PRGdb [33] and used to identify and classify putative resistance genes in Arabidopsis, eggplant, turkey berry, tomato and potato (Table 4), and the resistance gene distributions were plotted (Figure 1 and Additional file 1: Figure S1). This conservative approach revealed 336 resistance genes in Arabidopsis, including 44 CNL and 100 TNL class genes, which is comparable to results from domain prediction–based methods [36] in which 48 CNL and 89 TNL class genes were identified.

**Table 4:** **Summary of plant resistance genes in Solanum species and Arabidopsis**

	***A. thaliana***	***S. melongena*** L.	***S. torvum*** Sw.	***S. lycopersicum***	***S. tuberosum***
Total	336	621	815	505	774
CNL	44	110	194	99	219
TNL	100	46	66	29	93
RLK	102	221	255	134	156
RLP	19	84	128	77	132
TN	1	1	-	-	-
NL	-	16	21	41	46
Kinase	6	31	29	16	23
Other	64	112	122	109	105

CNL: N-terminal coiled coil–nucleotide-binding site–leucine-rich repeat, TNL: Toll interleukin1 receptor–nucleotide-binding site–leucine-rich repeat, RLK: receptor-like kinase, RLP: receptor-like protein.

Compared with Arabidopsis, each of the four *Solanum* species contained approximately twice the number of resistance genes, with 621 in eggplant, 815 in turkey berry, 505 in tomato, and 774 in potato. The wide intraspecific variation in number of resistance genes may underlie the species-specific differences in resistance to different types and quantities of pathogens and differences in the degree of responses to the same pathogen. The different resistance capability between eggplant and turkey berry may partly result from variation in the number of resistance genes, as turkey berry carries nearly 200 more resistance genes than eggplant. Resistance genes are frequently clustered in the genome—the result of both segmental and tandem duplications [36, 37]—and this was also observed in tomato (Figure 1, B1 ring) and potato (Additional file 1: Figure S1, B1 ring). Resistance genes also appeared to be clustered in eggplant (Figure 1, B2 ring and Additional file 1: Figure S1, B2 ring) and turkey berry (Figure 1, B3 ring and Additional file 1: Figure S1, B3 ring), but this observation needs verification with genome data.

Another difference between the *Solanum* species and Arabidopsis was the composition of resistance gene classes. TNL genes outnumbered CNL genes in the four *Solanum* species, which is similar to what has been observed in both grape and poplar (*P. trichocarpa*) but in contrast to what has been found in apple (*Malus domestica*), soybean (*Glycine max*) and Arabidopsis [38]. The CNL and TNL classes are the two major NL proteins, which are believed to act intracellularly [34], and the RLK and RLP classes are the two major membrane-localized receptor proteins that sense various pathogens and transduce signals to downstream intra- and intercellular networks [34]. The numbers of genes of all of these four classes were larger in turkey berry than in eggplant (Table 4). This may reflect amplification of the entire disease resistance pathway in turkey berry rather than duplication of a particular gene or class of genes to enhance pathogen defense and consequently improve fitness. The variation in the number of resistance genes was also evidenced by plotting the distribution of eggplant and turkey berry resistance genes against the tomato genome (Figure 1 B2 and B3 rings). As shown in Figure 1, the distribution patterns were similar (presence or absence) overall, but numbers of genes varied.

## Conclusions

Our results deepen our understanding of phylogenetic relationships, which will ultimately assist in eggplant improvement efforts. Furthermore, these high-quality unigenes will be useful in trait-related gene mining, as we demonstrated with the identification of plant resistance genes and comparison of these genes between species. Results from resistance genes identification indicated the high variation of resistance genes between them. In addition, these datasets can serve as reference transcriptomes for further analyses, such as quantitative gene expression profiling, to broaden our understanding of eggplant biology and to improve this agriculturally important vegetable.

## Methods

### Ethics statement

None of the species used in this study are endangered or protected, and all plants were grown in greenhouses, which complies with all relevant regulations. Therefore, no specific permits were required for the collection of samples.

### Plant materials and transcriptome sequencing

All samples of eggplant and turkey berry were collected from the experimental farm of the Department of Horticulture in Yangzhou University, Jiangsu Province, and were grown in pots containing peat, vermiculite and perlite (3:1:1, v/v) in a greenhouse at 28/18°C (12/12 h) day/night temperature with relative humidity ranging 70%–85%. For each species, the following tissues were sampled from seedling at the four true leaves stage: root, stem and young leaves. All samples were immediately frozen in liquid nitrogen and stored at −70°C for later use. The RNA extraction, library construction and RNA-Seq were performed at Beijing BioMarker Technologies (Beijing, China) following the protocol of Han *et al.*[39].

### Sequence data analysis and assembly

To obtain high-quality clean reads for transcript *de novo* assembly, the raw reads from transcriptome sequencing were filtered with the following criteria: (1) reads with adaptor contamination were removed, (2) low-quality reads were designated with “N” and (3) reads in which >10% of the bases had a Q-value < 20 were discarded. The clean reads were then assembled into contigs using Trinity [20] (http://trinityrnaseq.sourceforge.net/) with an optimized k-mer length of 31 for *de novo* assembly. Based on the paired-end information, the contigs (longer than 47 bp) were linked into transcripts. Finally, to eliminate redundant sequences, transcripts longer than 200 bp were clustered based on sequence similarities, and the longest transcript in each cluster represented the final assembled unigene that was subjected to functional and structural annotation.

### Evaluation of sequence and assembly completeness

Using TopHat2 [40] with default parameters, the clean sequencing reads from eggplant and turkey berry were aligned to the tomato and potato genomes. Tomato (ITAG2.3 release) and potato (PGSC DM 3.4 release) data were obtained from Sol Genomics Network (http://solgenomics.net/). The resultant accepted bam files were assessed for call depth at each nucleotide site using SAMtools [41], and the depth distribution was plotted for eggplant and turkey berry relative to the tomato and potato genomes.

The corresponding tomato and potato homologs of transcripts and unigenes of the eggplant and turkey berry were identified using BLASTX. Transcripts and unigenes were aligned with the parameters: −*evalue 1e-5 -outfmt 6 -max_target_seqs 1 -seg no*, and then the alignments were filtered for minimum alignment length of 50 amino acids and identity value of ≥30%. The distributions of eggplant and turkey berry unigenes and transcripts relative to the tomato and potato genomes were then plotted.

### Functional and structural annotation

To determine the functional categories of the unigenes, a BLASTX search with a cut-off E-value ≤ 10^5^ was performed against public protein databases, including the NCBI NR, SwissProt [42] and KEGG [43] databases and the potato (PGSC DM 3.4) and tomato (ITAG 2.3) protein sets. KEGG pathways were retrieved from the KEGG web server (http://www.genome.jp/kegg/) [44]. The output of the KEGG analysis includes orthology assignments and pathways that are populated with the orthology assignments. Domain-based alignments were carried out against the NCBI COG database [45] (http://www.ncbi.nlm.nih.gov/COG/) with a cut-off E-value of ≤ 1e^−5^. The resulting NR BLASTX hits were processed with BLAST2GO software [46] to retrieve the associated gene ontology terms with E-values ≤ 10^−5^ describing biological processes, molecular functions and cellular components [47].

The CDSs of each putative unigene were extracted according to the BLASTX results (homologous approach), with a minimum 150-bp cutoff value and the priority order of SwissProt, *Solanum* (tomato and potato) protein datasets and NR database if conflicting results were obtained. ESTSCAN software [48] was also used to determine the direction of sequences that did not align to any of the databases, and CDSs shorter than 150 bp were removed. To avoid missing potential coding transcripts, the unigenes for which CDSs were not predicted by either homologous or ESTSCAN approaches were subjected to an in-house script, which, like most gene prediction programs, uses fifth-order hidden Markov chains to model coding regions [49]. Again, the CDSs shorter than 150 bp were removed. The resultant CDSs extracted from the eggplant and turkey berry unigenes were translated into amino acid sequences with the standard codon table.

### Identification of gene orthologous groups

The translated eggplant and turkey berry amino acid sequences were pooled into a protein database with sequences (>50 amino acids) from another 11 plant species: *S. lycopersicum* (Sol Genomics Network ITAG2.3), *S. tuberosum* (Sol Genomics Network PGSC DM 3.4), *A. thaliana* (TAIR release 10), *C. papaya* (http://www.life.illinois.edu/plantbio/People/Faculty/Ming), *V. vinifera* (http://www.genoscope.cns.fr/externe/GenomeBrowser/Vitis/), *P. trichocarpa* (JGI release v2.0 annotation v2.2), *P. persica* (Phytozome v9.0), *C. sinensis* (http://citrus.hzau.edu.cn/orange/download/), *M. truncatula* (Medicago Genome Sequence Consortium release Mt 3.0), *Z. mays* (Maize Genome Project 5b.60 B73) and *O. sativa* japonica (MSU Release 7.0).

Self-to-self BLASTP was conducted for all amino acid sequences with a cut-off E-value of 1e^−5^, and hits with identity < 30% and coverage < 30% were removed. Orthologous groups were constructed from the BLASTP results with OrthoMCL v2.0.9 [21] using default settings.

### Phylogenetic tree reconstruction

Single-copy gene families were retrieved from OrthoMCL as described above and used for the following phylogenetic tree reconstruction steps. The families containing any sequences shorter than 200 amino acids were removed, the amino acid sequences in each family were aligned using MUSCLE v3.8.31 [50] with default parameters, and the corresponding CDS alignments were back-translated from the corresponding amino acid sequence alignments. The families were further filtered if the CDS alignment contained any taxon for which >50% of the data was missing. The remaining CDS alignments of each family were separated into three sets corresponding to each of the three codon positions. The four supermatrices (all codon positions and each codon position) were then separately assembled into supergenes using an in-house Perl script. The refined supergene data were then subjected to maximum likelihood phylogenetic analyses using PhyML3.1 [25]. The HKY85 + gamma substitution model was selected, and bootstrap values were calculated using the aLRT model (parameters: −*d nt –m HKY85 –b −4 –a e -c 4*). TreeBeST (version 1.9.2, http://treesoft.sourceforge.net/) was used to root the trees if necessary.

### Estimation of divergence time

Two datasets were generated from the CDS alignments used for divergence time estimation: (1) a dataset containing the first two partitions, the first and second codon positions of the sequences; and (2) a set containing all the three partitions corresponding to all the three codon positions in the sequences. Divergence times were estimated under a relaxed clock model in the MCMCTree program in the PAML4.7 package [28], with “Independent rates model (clock = 2)” and “JC69 model” selected for our calculations. The MCMC process performs 40,000 iterations after a burn-in of 15,000 iterations. Other parameters were the default settings of MCMCTree. We ran the program twice for each dataset to confirm that the results were similar between runs. The following constraints were used for time calibrations:

(i) 140–150 Mya, monocot–dicot split [51]
(ii) 94 Mya, lower boundary for *Vitis*–Eurosid split [52]
(iii) 68–76 Mya, Caricaceae–Brassicaceae split [30]
(iv) 44 Mya, upper boundary for the Solaneae [53]
(v) 5.1–7.3 Mya, tomato–potato split [2, 31]

### Identification of plant resistance genes

Amino acid sequences for 112 reference resistance genes were downloaded from the Plant Resistance Genes database (PRGdb; http://prgdb.crg.eu/wiki/Main_Page) [33]. BLASTP was used to identify and classify putative resistance genes in eggplant, turkey berry, tomato potato and Arabidopsis (parameters: −*evalue 1e-5 -outfmt 6 -max_target_seqs 1*). By parsing tabular outputs using in-house PERL scripts, results were filtered with a threshold cut-off of 40% identity and 50% coverage, and then homologous sequences were extracted and classified.

### Data availability

The sequences reported in this paper have been deposited in the National Center for Biotechnology Information (NCBI) Sequence Read Archive (SRA) and Transcriptome Shotgun Assembly (TSA). Raw paired-end reads are available through the NCBI SRA under accession numbers [SRA: SRR1104129] (eggplant) and [SRA: SRR1104128] (turkey berry). Transcripts are available through the NCBI TSA under accession number GBEF00000000 (eggplant) and GBEG00000000 (turkey berry).

## Electronic supplementary material

Additional file 1: Figure S1: Distributions of genomic elements of potato eggplant and turkey berry on potato genome. **Figure S2**: Distributions of depth of reads and densities of genes on tomato genome. **Figure S3**: Distributions of depth of reads and densities of genes on potato genome. **Figure S4**: Maximum likelihood trees based on 276 single-copy genes. **Figure S5**: Estimation of divergence time using the first and second codon positions. (DOCX 2 MB)
