# Supplementary material for: Comparative transcriptome analysis of eggplant (Solanum melongena L.) and turkey berry (Solanum torvum Sw.): phylogenomics and disease resistance analysis
Source: BMC Genomics. 2014 May 31;15(1):412. doi: 10.1186/1471-2164-15-412 (PMC4070557; doi:10.1186/1471-2164-15-412)
Supplement: Supplementary file 1 — Additional file 1: Figure S1: Distributions of genomic elements of potato eggplant and turkey berry on potato genome. Figure S2: Distributions of depth of reads and densities of genes on tomato genome. Figure S3: Distributions of depth of reads and densities of genes on potato genome. Figure S4: Maximum likelihood trees based on 276 single-copy genes. Figure S5: Estimation of divergence time using the first and second codon positions. (DOCX 2 MB) [file 12864_2014_6131_MOESM1_ESM.docx]

**Additional file 1.**

Figure S1 - Distributions of genomic elements of potato eggplant and turkey berry on potato genome.

Figure S2 - Distributions of depth of reads and densities of genes on tomato genome.

Figure S3 - Distributions of depth of reads and densities of genes on potato genome.

Figure S4 - Maximum likelihood trees based on 276 single-copy genes.

Figure S5 - Estimation of divergence time using the first and second codon positions.

**
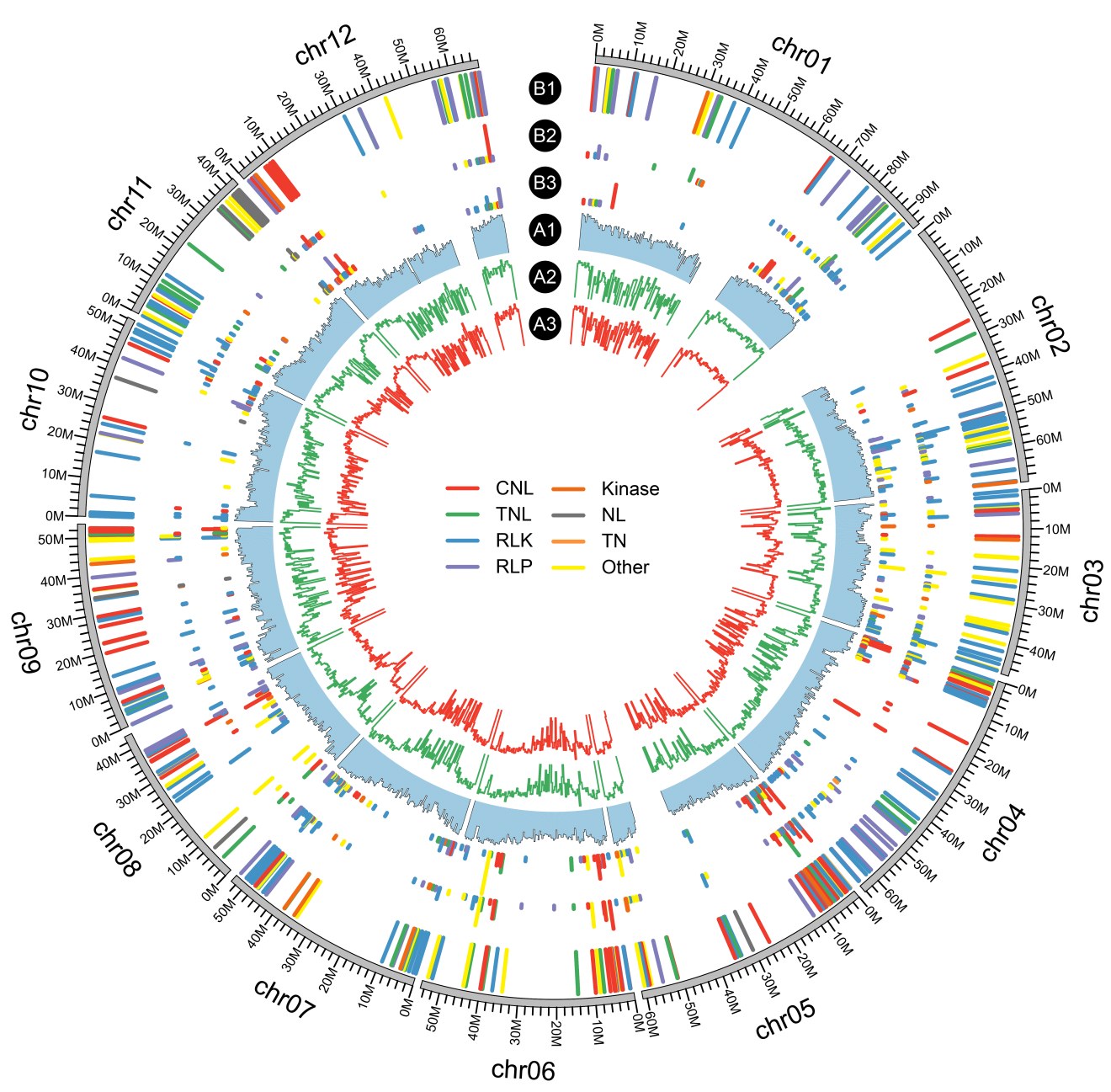
**

**Figure S1. Distributions of genomic elements of potato eggplant and turkey berry on potato genome. A1**, The log2-transformed potato gene density (blue histogram ring) along the potato chromosomes (chr, outer ring). Gene density represented as number of genes per 500 kb (non-overlapping, window size = 500 kb), and the log2-transformed gene density ranged from 0.00 to 6.50. **A2–A3**, The log10-transformed average depth of RNA-Seq reads from eggplant (**A2**, green histogram ring) and turkey berry (**A3**, red histogram ring). We used the 500kp non-overlapping sliding windows to calculated the average depth, and the log10-transformed average depth ranged from 1.50 to 6.50. **B1**, Potato resistance genes. Colors correspond to the gene product types indicated in the center of the diagram. **B2** and **B3**, resistance genes of eggplant (B2 ring) and turkey berry (B3 ring). The square root of the number of resistance genes per potato homolog (BLASTX hits) ranged from 1.00 to 3.00 (for illustration purposes, the minimum was set at 0.80).


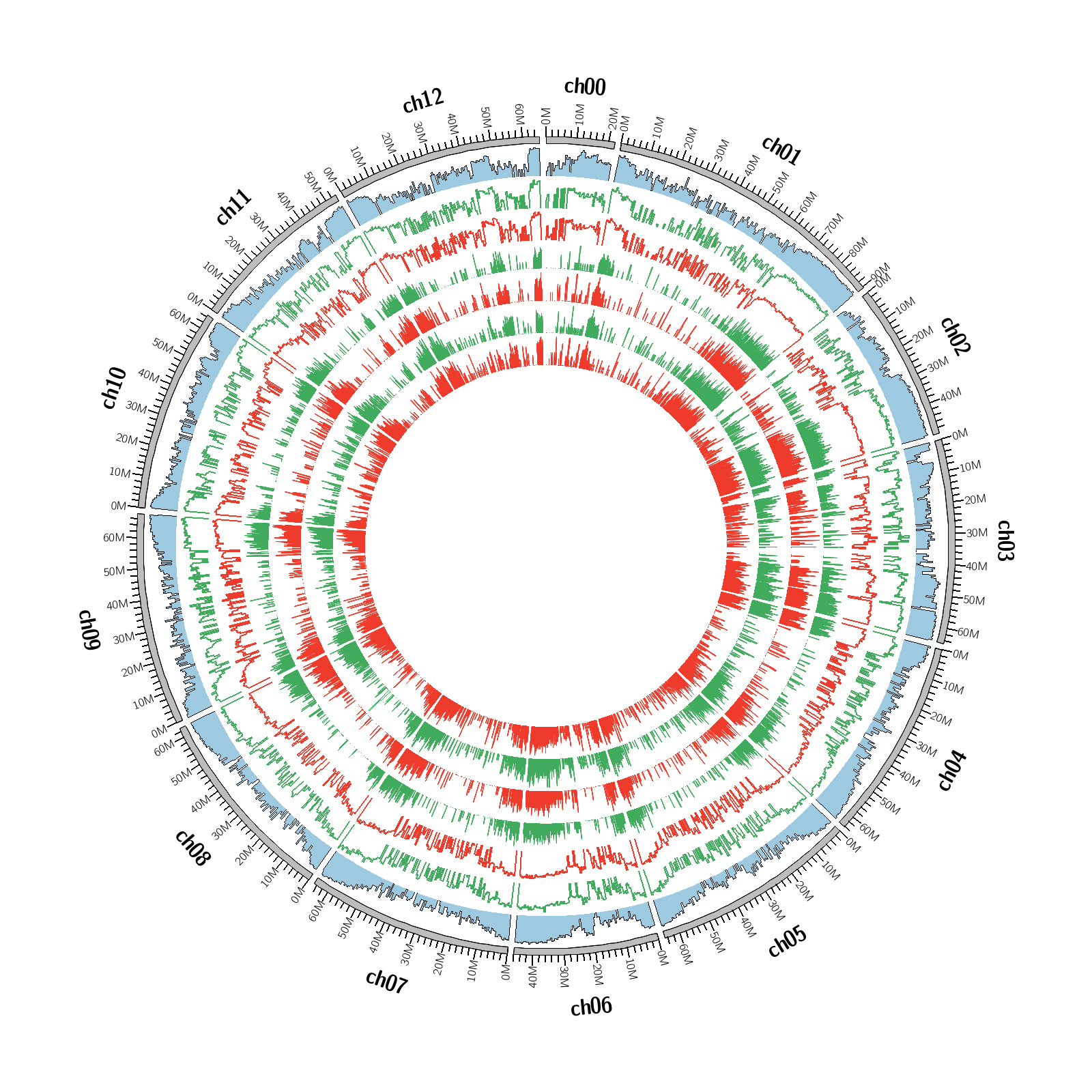


**Figure S2. Distributions of depth of reads and densities of genes on tomato genome.** The outer three rings, from outermost to innermost, are the log2-transformed tomato gene density (blue histogram) and the log10-transformed RNA-Seq depths of eggplant (green) and turkey berry (red), which are the same as the rings of **Figure 1** A1–A3 respectively. The next two rings are eggplant (green) and turkey berry (red) unigene densities (log2-transformed, ranging from 0.00 to 3.50). The innermost two rings are eggplant (green) and turkey berry (red) transcript densities (log2-transformed, ranging from 0.00 to 4.50).


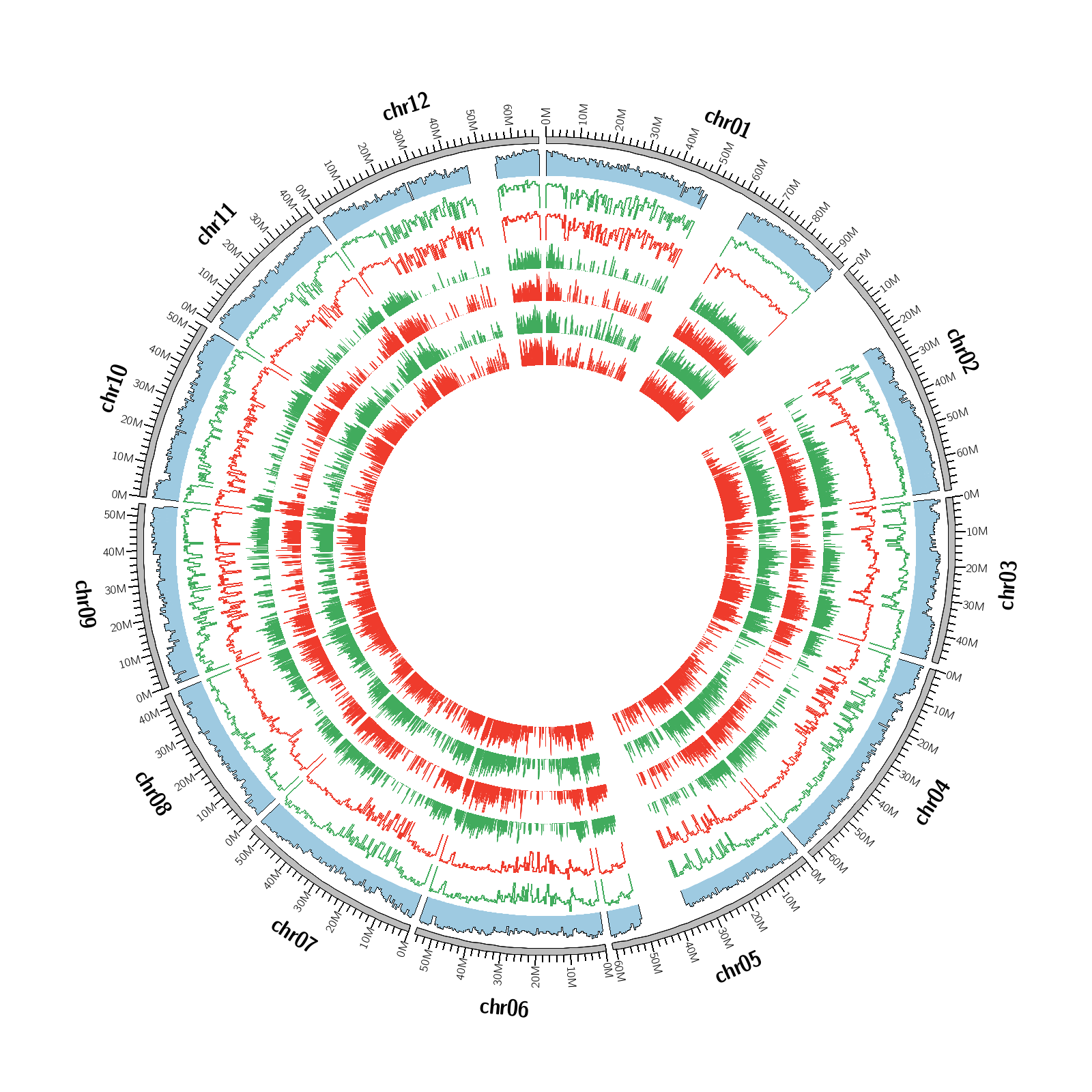


**Figure S3. Distributions of depth of reads and densities of genes on potato genome.** The outer three rings, from outermost to innermost, are the log2-transformed potato gene density (blue histogram) and the log10-transformed RNA-Seq depths of eggplant (green) and turkey berry (red), which are same as the rings of **Figure S1** A1–A3 respectively. The next two rings are eggplant (green) and turkey berry (red) unigene densities (log2-transformed, ranging from 0.00 to 3.50). The innermost two rings are eggplant (green) and turkey berry (red) transcript densities (log2-transformed, ranging from 0.00 to 4.50).


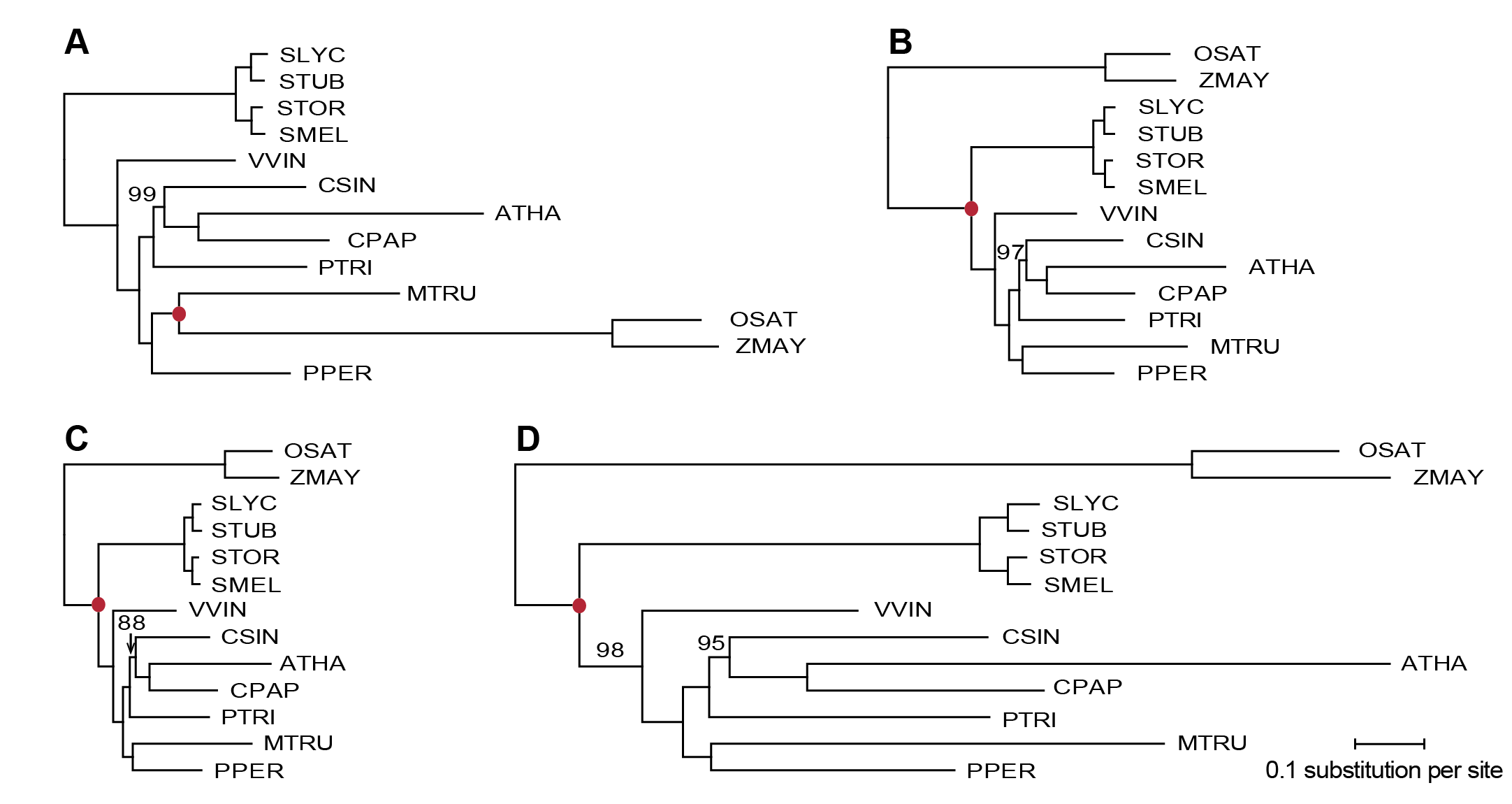


**Figure S4. Maximum likelihood trees based on 276 single-copy genes.** Maximum likelihood trees obtained from the full-length CDSs (**A**), the first codon positions (**B**), the second codon positions (**C**) and the third codon positions (**D**). The branch lengths of all the trees are scaled to 0.1 substitutions per site (scale bar bottom right). The abbreviations on each leaf are the same as those in Figure 4, and red dots on the tree indicate where the monocot clade was placed. The bootstrap values less than 100% are indicated on each tree.


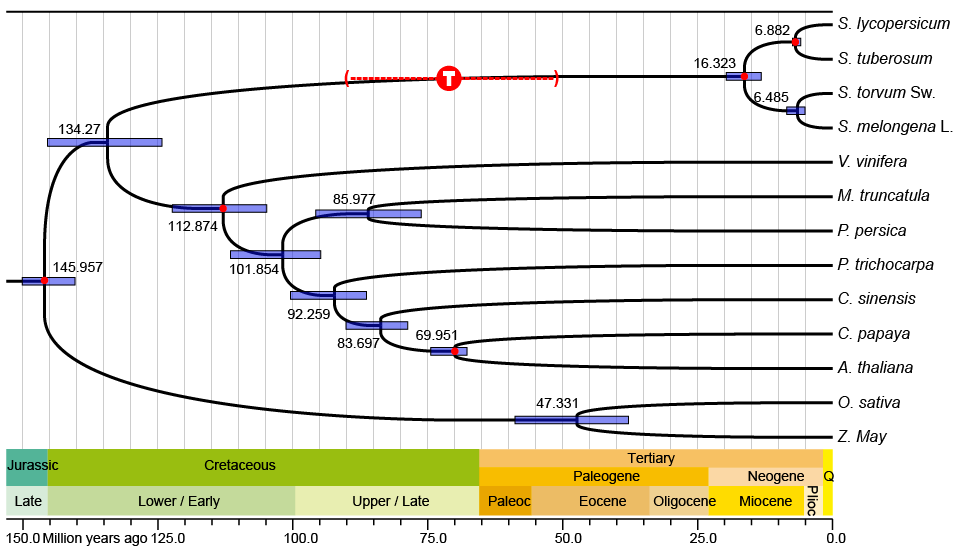


**Figure S5. Estimation of divergence time using the first and second codon positions.** The purple bars at the nodes indicate 95% posterior probability intervals. The geological time scale is in millions of years. The red dots correspond to the calibration time points listed in the **Materials and Methods**. Confirmed whole-genome triplication shared by *Solanum* and estimated at 71 (±19.4) MYA[2] is shown with annotated circles (‘T’), with dashed line indicating confidence interval. Paleoc: Paleocene; Plioc: Pliocene; Q: Quaternary.
